# How are competency frameworks perceived and taught? An exploratory study in the context of pharmacy education

**DOI:** 10.1007/s40037-018-0432-y

**Published:** 2018-04-27

**Authors:** Elise Paradis, Rebecca Zhao, Jamie Kellar, Alison Thompson

**Affiliations:** 1 Leslie Dan Faculty of Pharmacy, University of Toronto, Toronto, ON Canada; 2 Faculty of Arts and Sciences, University of Toronto, Toronto, ON Canada

**Keywords:** Competency frameworks, Faculty perspectives, Curricular reform, Qualitative research

## Abstract

**Introduction:**

Faculties of Pharmacy worldwide have to adapt their curriculum to accreditation criteria. The present study explored how the Association of Faculties of Pharmacy of Canada’s (AFPC’s) 2010 Educational Outcomes are perceived and taught at the Leslie Dan Faculty of Pharmacy (LDFP). These outcomes were adapted from the CanMeds Physician Competency Framework which describes both medical expert and non-expert roles.

**Methods:**

We wondered if pharmacy would struggle, as medicine has, to integrate these roles into curricula in meaningful ways, given the absence of previous studies from Pharmacy. We conducted an exploratory interview study with 10 core faculty members in charge of courses where non-expert roles were taught. Data were analysed using conventional content analysis.

**Results:**

Faculty members understood that the AFPC Outcomes are important for students, patients, and the profession of pharmacy, and some saw the roles as knowledge-based and teachable using content from academic disciplines. However, most saw them as skills taught informally or through clinical experience. They used the roles as a framing device to legitimize their course content and relied on informal role modelling to do most of the teaching. The few faculty members who taught content related to these roles had postgraduate education in a social science or humanities discipline.

**Discussion:**

Similar to studies of Faculties of Medicine, our study highlights the difficulty of translating a role-based, competency framework into concrete, integrated curricula for students. Competency development should be explicitly embedded into the curriculum and cannot be left to individual instructors.

## What this paper adds

Many educational reforms are rolled out from the top down, and the introduction of competency frameworks such as the CanMEDS or ACGME frameworks has followed this pattern. Our exploratory study in a faculty of pharmacy in Canada shows that the adoption may have been both top down and bottom up as pharmacy responds to external threats to the profession as its scope of practice expands into prescribing. Despite buy-in from faculty members around the competency framework, their ability to integrate competency frameworks into their teaching was limited by a lack of common language and understanding of the roles or competencies. Faculty development will be essential.

## Introduction

In 2005, the Royal College of Physicians and Surgeons of Canada published the CanMEDS framework, inspiring a major medical education reform in Canada and around the world. Indeed, the CanMEDS framework is now ‘one of the world’s most widely used competency frameworks’ [1]. Given the centrality of physicians in the Canadian healthcare system, it is not surprising that other health professions—including pharmacy—have taken up the CanMEDS roles and used them as a guide to orient their own professional education standards.

The role of the pharmacist has changed significantly over the past decades, and professionals once responsible for compounding and dispensing medication are now repositioning themselves as patient care providers [2]. This shift is evidenced by changes in pharmacy education from the BScPharm to the PharmD degree [2, 3] and expanding scopes of practice [3]. In particular, the new PharmD degree marks an explicit education movement in North America towards the identification of pharmacists as clinicians with education and titles similar to those of physicians, and with a desire to produce confident, competent graduates who are able to effectively contribute to patient-centred services [3].

The first educational outcomes document for the PharmD degree in Canada was published by the Association of Faculties of Pharmacy of Canada (AFPC) in 1999, and built upon the previous BScPharm requirements to specify five ‘Outcome Units’ and expected levels of competence [4]. It was replaced in 2010 by the *Educational Outcomes for First Professional Degree Programs in Pharmacy*, the educational framework that was in use during the course of our study [5]. The Task Force charged with the development of the 2010 *Outcomes* sought to concretize the competencies needed for pharmacy graduates to be patient-centred Medication Therapy Experts into seven ‘roles’ (see Table 1) that parallel the 2005 CanMEDS roles, recognizing that the ‘adoption of a common format and language across the professions would support collaboration and inter-professional care’ [5].

**Table 1:** Educational outcomes and their definitions

*Care provider*	Pharmacy graduates use their knowledge, skills and professional judgement to provide pharmaceutical care and to facilitate management of patient’s medication and overall health needs
*Communicator*	Pharmacy graduates communicate with diverse audiences, using a variety of strategies that take into account the situation, intended outcomes of the communication and the target audience
*Collaborator*	Pharmacy graduates work collaboratively with teams to provide effective, quality healthcare and to fulfil their professional obligations to the community and society at large
*Manager*	Pharmacy graduates use management skills in their daily practice to optimize the care of patients, to ensure the safe and effective distribution of medications, and to make efficient use of health resources
*Advocate*	Pharmacy graduates use their expertise and influence to advance the health and well-being of individual patients, communities, and populations, and to support pharmacist’s professional roles
*Scholar*	Pharmacy graduates have and can apply the core knowledge and skills required to be a medication therapy expert, and are able to master, generate, interpret and disseminate pharmaceutical and pharmacy practice knowledge
*Professional*	Pharmacy graduates honour their roles as self-regulated professionals through both individual patient care and fulfilment of their professional obligations to the profession, the community and society at large

Adapted from the AFPC (2010) *Educational Outcomes*

While medicine has fully adopted CanMEDS, it is unclear whether and how other professions have embraced their own versions of the framework. Exactly how to integrate the roles remains unclear in all disciplines, and the literature currently provides no guidance on this weighty issue.

In the broader context of education reform, these questions take on added significance when such endeavours have often been found to ignore the epistemic, social, and systemic contexts of curricular reform [6].

## Objective

Our study aimed to answer the following question: How have the revised 2010 AFPC roles been included in the curriculum at the Leslie Dan Faculty of Pharmacy (LDFP) at the University of Toronto? An exploratory study was warranted given the absence of previous studies on the integration of the AFPC *Educational Outcomes* in the curriculum of faculties of pharmacy. Indeed, a librarian-assisted search yielded no published articles on the AFPC *Outcomes*, despite several studies published in the context of medicine reporting that the teaching and assessment of the CanMEDS seven roles is often wavering, inadequate, and mostly implicit [7–9].

## Methods

The present study is nested within the CanMEDS Knowledge Project at the University of Toronto, a project that seeks to understand how the social science and humanities knowledge underpinning the non-medical expert CanMEDS roles (and their correlates in other health professions such as the AFPC Outcomes) is implemented in different systems, institutions, and curricula, and that seeks to support educators who wish to do so [10, 11]. The interview script used in this study was developed by the CanMEDS Knowledge Project team, then adapted by EP and AT for the pharmacy context. It focused on: exploring participants’ understanding of the roles, their significance for students, patients and the profession, teaching strategies and specific examples of how the roles are being addressed in their courses, their perspectives on the *Outcomes* and their import to the curriculum, how the *Outcomes* are perceived by their colleagues and students, and how they might contribute to interprofessional education. All courses in the formal curriculum used the 2010 *Educational Outcomes *as a curricular map, and thus competency-based education forms the backbone of the curriculum. The study focused on recruiting instructors who were involved with curricular and course design for courses where the non-expert roles were identified as being educational outcomes in the newly created PharmD program. The courses that focus on non-expert *AFPC Educational Outcomes* are both practice-based and didactic, and classroom and laboratory teaching occur within the first three years of the four-year, PharmD program. The forms of assessment employed in these courses run the gamut from essays, group presentations, formal written examinations, to simulated practice-based assessments. The fourth year is comprised entirely of experiential education, and preceptors mentor students in their rotations. There are also shorter experiential summer rotations within the curriculum which assess professionalism and clinical skills.

Three authors—EP, JK, and AT—are faculty members at the LDFP, while RZ was an Arts and Science student at the University of Toronto. Ethics approval was obtained from the University of Toronto Research Ethics Board (protocol reference # 31765) before recruitment and data collection. Potential participants were recruited via email and given detailed background information on the study. To get a broad portrait of faculty members’ perspectives on the current pharmacy curriculum and how it incorporates the AFPC roles, the 10 faculty members who are the core group responsible for course development and delivery of courses in the undergraduate curriculum were selected and interviewed by RZ, AT and EP in the summer and fall of 2016.

All of the faculty members invited to participate in the study were interviewed. Characteristics of the sample are not given to protect the anonymity of participants.

Interviews lasted between 32 to 64 minutes, with an average duration of 47 minutes. They were recorded, transcribed and de-identified by RZ, reviewed for accuracy by EP, and imported into MAXQDA Version 12 for analysis. Original emphasis during interviews is in regular font in the italicized quotes cited below. Data were analysed following Hsieh and Shannon’s conventional content analysis ([12]; see Table 2). While RZ and EP did most of the coding and analysis, the authorial team met regularly to discuss study progress, themes and coding, and to review the article.

**Table 2:** Steps in conventional content analysis

Step	Description	Author in charge
1	Conduct interviews with open-ended questions and have probes that are also open-ended or specific to participant comment	RZ, EP, AT
2	Repeatedly read all data from beginning to end	RZ, EP
3	Read texts word by word for coding and highlight exact words to capture key concepts and thoughts	RZ, revised by EP
4	Make notes of first impressions, thoughts, and initial analyses. Codes derived from the text will become the initial coding scheme	RZ and EP
5	Sort codes into categories based on how codes are related to each other. Categories are organized into meaningful clusters	EP
6	Organize categories into a hierarchical structure by breaking down categories into subcategories	EP
7	Develop definitions for each category, subcategory, and code. Identify relationships between categories	EP, RZ, JK, AT
8	Write about relevant theories and findings in the Discussion of the paper	RZ, EP, JK, AT

Adapted from Hsieh and Shannon (2005) [12]. Three approaches to qualitative content analysis

## Results

In this section we discuss the main categories we developed in our analysis of faculty members’ transcripts: the importance of roles, the perception of roles, and the modalities and depth of teaching the roles.

### Importance of roles

All faculty members in our sample saw the roles as extremely important, and articulated their importance on three levels: for students, for patients, and for the profession of pharmacy.

#### For students

Most faculty members saw the roles as key to students’ professional development. When asked how important they perceive these roles to be, one faculty member answered emphatically, ‘*It is essential because they were developed to be the clinicians in the 21st century’* (F6). Success as a pharmacist was also seen as hinging upon the roles. For others, the non-expert roles supplement pharmaceutical knowledge and expertise: ‘*I think it’s for the well-rounded professional, these are the characteristics … competencies, that you really need to have’* (F5). The Faculty was thus positioned as having a strong mandate to educate ‘well-rounded’ professionals who can enact the AFPC roles.

#### For patients

The importance of the roles to patient care and interactions with patients was sometimes invoked by faculty: ‘*In the absence of these competencies or roles, I’m not sure that the quality of patient care would be what we need [it] to be … It becomes a transaction. Just a transaction—a clinical one—in absence of them’* (F6).

For another faculty member, the attainment of these roles would help pharmacists legitimize their expertise to patients in the face of easier access to information for patients via the internet: ‘*[T]here’s *so* much information on the Internet now, that any patient … can find information about how to diagnose and treat any illness they want … But they hopefully can also appreciate that they need to get some *expert* advice for things’* (F2).

For this interviewee, the AFPC roles also allow pharmacists to maintain their professional authority as knowledgeable medication experts at a point in time where information is cheap and readily available online, but not necessarily correct or helpful for patients.

#### For the profession

The importance of the roles for the profession of pharmacy was sometimes articulated as arising from a need to show the value that pharmacists bring to the healthcare system: ‘*Well, I think it’s quite important because I think together they help to maintain the, I guess the ability of the profession to add value to the health care system’ *(F2).

Another faculty member described the need for pharmacy to protect and expand its scope in the face of external threats to their authority:

*I think if pharmacy wants to really keep going down this road of patient care and expand the scope of practice that they have now, they really are going to have to be able to do this well … because they’re going to be increasingly under threat, I think, from a number of fronts and I think they need to be able to demonstrate that they can bring something that a physician can’t or nurse can’t or a nurse practitioner *(F9).

In a system where different professional groups need to legitimize their role and scope, the AFPC roles provide a necessary foundation for professional care that goes beyond expert knowledge of medications, and allow pharmacists to position themselves as having a place in the system that is not easily replaced by the internet or other professional disciplines.

### Perception of the roles

Faculty members shared their perspectives when asked how they teach the AFPC roles. We grouped their perceptions of roles into two main categories: roles *as knowledge*, and *roles as skills*.

#### Roles as knowledge

The view of the roles as knowledge-based was quite rare. The faculty members who discussed these roles as anchored in knowledge justified their view by underscoring the value of disciplinary knowledge for the pharmacy curriculum: ‘*Yes, they’re knowledge-based and yes, they can be taught … it’s got to be more content-based because there is a* wealth* of knowledge out there that we can impart or create’ *(F9). For this faculty member, the curriculum’s implicit teaching of roles through experiential education or role modelling by faculty members and preceptors is insufficient to develop students’ understandings of the roles. Another faculty member elaborated a similar vision for the Communicator role:

*So within each of these competencies, there actually is a scholarly foundation. There is a rich tradition in disciplines like psychology, and sociology, and anthropology. Communication; there is actually a* science *of communication. … We tend to teach communication as a series of stimulus-response techniques. … As a university-based program, it should be possible to actually teach communication as a discipline* (F1).

Here, the participant seems to be invoking the importance of the university context to the program, perhaps drawing on the distinction between university-based programs which draw on research and disciplines within the academy, and technical programs which are focused on imparting vocational skills.

#### Roles as skills

Most faculty members saw roles as the demonstration of internalized skills that arise from emulating behaviour and integrating them with clinical practice, as opposed to having materials that aim to impart knowledge related to the roles: ‘*It’s not about materials for me, it’s about understanding the concept of what [the role] means to them as a developing professional … What’s *important* is that they model this behaviour, make this behaviour part of their practice’* (F6).

Another faculty member identified a problem, however, when skill development occurs without understanding of the connection to the roles and why they matter: ‘*I don’t know that it’s materials that they need as much as that there needs to be transparency about *why* we’re doing what we’re doing’* (F10). The teaching of skills without connecting them to the roles is seen as sub-optimal for learning. However, materials designed to impart knowledge about the roles were not perceived to be as important to achieving the educational outcomes as developing active or experiential learning strategies to aid in the development of skill proficiencies.

### Modalities and depth of teaching

Different faculty members taught the roles using different modalities and in varying depth. For some, the roles were framing devices for their course; for others, roles were taught either through role modelling, through exposure, or as knowledge.

#### AFPC roles as framing device

Some participants mentioned using the roles to situate their course within the broader curriculum, as was mandated at the time the courses were developed for the PharmD curriculum. One faculty member talks about them in their introductory lecture and makes students reflect on the roles (F6). Another suggested that they are ‘*trying to use these, I guess, as a framing message at the beginning and the end of a rotation to kind of hopefully be able to let the learners know that we got a framework that hopefully holds it together*’ (F2). This same faculty member admitted, however, that she and her colleague use only ‘*one or two slides*’ to describe the roles, and that they ‘*really don’t emphasize it much, just to say that these rotations are created with course outlines, have been mapped to the competencies*’ (F2).

#### Teaching roles through role modelling

One key teaching modality described by several faculty members—all of them practising pharmacists—was role modelling. One faculty member suggested: ‘*I would try to model certain kinds of attributes and probably the one that I would try to model the most would be Scholar*’ (F1). For another, ‘*a lot of it with communication is the modelling’* (F7). Talking about the Professional role in particular, one faculty member shared: *‘In class, I don’t know if we actually have anything specific other than to be role modelling what we do. I think it’s more role modelling in that [it] might occur as much out of class as it does in class’* (F8).

For many, modelling was fundamental to the teaching of these roles, and other forms of teaching were described as less valuable. By ‘*practising what you’re preaching*’, they said, you avoid the pitfalls of ‘*just sitting here as a book-learned person and then you can’t really speak to it*’ (F7).

#### Teaching roles through exposure/experience

It is unsurprising, given the dominant framing of the AFPC roles as skills, that our discussions of modalities of teaching often discussed the importance of the experiential rotations (1^st^ year Early Practice Experiences or EPEs, or 4^th^ year Advanced Pharmacy Practice Experience [hereafter APPEs]) as major enablers of the acquisition of the roles. Having the experience of ‘being in the actual practice and having real patients’ is seen as making the teaching of these roles less abstract and more real (F7). One faculty member, in reference to teaching communication shared, ‘*It’s really hard until you actually are doing it. I mean, how would I do that in my course?’* (F5).

Some faculty members decried how the curriculum assumes that the roles are learned in the first three years, and then the rotations are designed to assess the roles in action:

[*W]e make some assumptions, I think, when the students are coming to us at the end of third year, that they’ve already been hit by these terms all the way along and so we don’t typically go into a lot of detail, we just kind of say, ‘Remember these AFPC outcomes? Well here they are again and here’s how we apply them out in the practice now instead of the classroom’* (F2).

This is problematic since it assumes pre-existing skills and knowledge that might actually be lacking, given the emphasis on developing the role-based competencies through clinical experience and modelling by faculty which occurs largely outside of the classroom context.

#### Teaching roles as knowledge/materials

As we have seen above, most faculty members see the roles as skills-based rather than knowledge-based. Exceptions arose in interviews when disciplinary-trained faculty members (e. g. psychology, economics, sociology or ethics) shared examples of how they bring materials, concepts and insights from their disciplinary background into their teaching and discussed how these foundations allows them to go beyond what is typically perceived as the core curriculum in pharmacy.

## Discussion

The publication of the 2005 CanMEDS framework has led to major health professions educational reforms in medicine, nursing, pharmacy and beyond. It is possible to view the AFPC’s 2010 *Educational Outcomes *as being the result of external pressures on the profession, as has been the case in medicine, rather than the result of a fully self-driven, internally motivated process. However, it is also possible to speculate on the basis of some participant views that the adoption of the CanMEDS approach to professional competency could have been strategic on the part of the AFPC, in that it positions pharmacy as a profession that requires the same basic competencies as medicine at a time when pharmacist prescribing is expanding the scope of pharmacy practice into what has traditionally been medicine’s territory. It has also been argued that the non-expert roles have a protective function, armouring the profession against threats to its medical (or in this case, pharmaceutical) authority [13]. We see this reflected in these data where participants discuss how the roles protect against threats to the profession and how they provide legitimacy to pharmacists’ contributions to the healthcare system. The protective function, together with the legitimizing effect of embracing (an adapted) *medical* competency framework in the context of pharmacy’s broadening prescribing authority, may well explain why all faculty members interviewed viewed the roles as important for the profession.

Faculty members are not unified in their views of the roles, however. Most of our participants see the roles as skills and develop them through role modelling and experiential exposure. Some faculty members expressed beliefs that academic, ‘book’ knowledge is much less useful and valuable than experiential knowledge. In contrast, only three of the faculty members we interviewed teach the roles using a knowledge base, anchored in their academic discipline of origin. Our exploratory study describes a small sample of instructors, but it raises important concerns about the future of initiatives such as the CanMEDS Knowledge Project, and its aim to apply the wealth of knowledge from the disciplines that have expertise in the non-expert roles [10]. It is difficult to see how this can occur when many pharmacy educators focus on practice proficiencies and skills, and not substantive knowledge of the disciplines that underpin these non-expert roles.

It is slightly more concerning—if not surprising—that concrete examples of how the AFPC roles are taught in our curriculum were very rare. Simply adding slides describing the roles to frame courses will likely be insufficient for students to internalize them. Moreover, ‘role modelling’ has some limitations: its efficiency and efficacy are very difficult to evaluate, and its success depends on students being aware of it and on instructors being skilled in the specific roles they are aiming to model [14]. It is unclear, then, what our students are learning in the informal curriculum with respect to the AFPC roles, and whether our curriculum enables them to enact their full professional scope. Given the dominance of biomedical knowledge in health professional faculties [11], however, we were not surprised that limited attention has been given to the disciplinary ‘book’ learning that underpins the non-expert AFPC roles in the development of the curriculum at the Leslie Dan Faculty of Pharmacy to date.

Medicine is further along in its scholarship around competency frameworks than pharmacy, but our research echoes previous findings of limited teaching and limited evaluation of the seven roles in medical education curricula [9, 15]. Furthermore, our finding that faculty cannot articulate explicitly how they teach the roles is echoed in the literature from other disciplines who use competency frameworks [14, 16]. More broadly, our findings on faculty perspectives reflect the different epistemological understandings of competency-based frameworks, as well as the ambiguity about the relationship between roles and competencies that others have noted elsewhere [7]. We believe that our research will resonate with faculty members in other health professions faculties who still struggle with the implementation of competency frameworks, both in North America and abroad, regardless of whether they are adopted because of internal or external pressures on the profession.

Our exploratory study shares the limitations of most small-size qualitative studies: when considered through positivistic standards, it is unlikely to be broadly generalizable. We believe, however, that anchoring of our findings in educational theory will allow readers to compare their own contexts with the one we have described here. Moreover, the fact that the project leaders (EP and AT) were colleagues of the faculty members that were interviewed might have influenced what they were comfortable sharing. The interviews conducted by RZ, however, an undergraduate student from the Faculty of Arts and Sciences, did not differ substantively from those conducted by EP. Further research should study the curricular materials related to competency frameworks, the perspectives of students on their learning of the roles and on curricular delivery methods, as well as the perspectives of faculty members with backgrounds in the basic and applied sciences. Comparative international research might also shed light on cultural differences in interpretation and translation of competency frameworks.

As noted recently by Rachel Ellaway, competency frameworks are ‘tools of consensus, coordination, or control’ [17], and should be studied as such. The 2015 CanMEDS competency framework is still being rolled out, and the AFPC released the next version of its *Educational Outcomes* in 2017, which connects roles, competencies, and key concepts [18]. As we move forward with the implementation of these frameworks, it seems of paramount importance for educators to come together as a group to define how—which modalities, and in what depth—they will be teaching the roles and competencies; to build consensus, coordinate, and control their future. While faculty members generally see competency frameworks as important, answering questions as to their nature—are they knowledge, skills, or both?—and the optimal way to teach them—role modelling, experience, disciplinary knowledge, or all of the above?—will be fundamental to their success. Leaving it to individual instructors to incorporate them into their teaching will likely not lead to optimal training for the next generation of health professionals. Engaging educators, students and leadership in curriculum mapping, prioritizing, integration and assessment will play a major role in strengthening the delivery and thus the learning of the roles, and thus in educating the health professionals we want for the future.
